# Compassionate use of experimental therapies: who should decide?

**DOI:** 10.15252/emmm.201505262

**Published:** 2015-07-22

**Authors:** Patricia J Zettler

**Affiliations:** Center for Law, Health & Society, Georgia State University College of LawAtlanta, GA, USA

## Abstract

In addition to being an example of unsubstantiated hype about regenerative medicine, the controversy around the Italy-based Stamina Foundation's unproven stem cell therapy represents another chapter in a continuing debate about how to balance patients' requests for early access to experimental medicines with requirements for demonstrating safety and effectiveness. Compassionate use of the Stamina therapy arguably should not have been permitted under Italy's laws, but public pressure was intense and judges ultimately granted access. One lesson from these events is that expert regulatory agencies may be the institutions most competent to make compassionate use decisions and that policies should include more specific criteria for authorizing compassionate use. But even where regulatory agencies make decisions based on clear rules, difficult questions will arise.

Earlier this year, a 6-year saga about a much hyped stem cell-based treatment for neurological diseases came to a close when criminal charges against the founder of the Italy-based Stamina Foundation, including charges of fraud, were resolved through a plea bargain. Founded in 2009, the Stamina Foundation claimed that it was transforming mesenchymal stem cells from bone marrow into neural stem cells and that injections with these cells would be a treatment for various neurodegenerative diseases, including Parkinson's disease and muscular dystrophy (Cattaneo & Corbellini, 2014). Their claims were scientifically implausible and unsupported by published evidence, but, understandably, convinced numerous people with devastating diseases and few treatment options to seek access to the therapy (Rial-Sebbag & Blasimme, 2014). Before the “compassionate use” of Stamina's “therapies” was stopped, more than 100 patients had received it, with many paying thousands of Euros (Rial-Sebbag & Blasimme, 2014).

it is critical for public health that the law requires that new medicines are shown to be safe and effective before they can be marketed.

The popular demand for the Stamina stem cell therapy, despite the lack of any evidence supporting its safety or efficacy, serves as a vivid example of both the appeal and the dangers of making unsubstantiated claims about regenerative medicine. Beyond this, the Stamina controversy represents another chapter in a longstanding debate about how to balance seriously ill patients' desire to use experimental medicines—what the European Medicines Agency (EMA) calls “compassionate use,” and the US Food and Drug Administration (FDA) calls “expanded access”—with legal requirements for demonstrating safety and effectiveness before clinicians can use these medicines (Zettler & Greely, 2014). On the one hand, it is critical for public health that the law requires that new medicines are shown to be safe and effective before they can be marketed. On the other hand, seriously ill patients may not have the time to wait for months or years until a therapy has been approved. This tension gives rise to compassionate use controversies.

Patients have repeatedly been enticed to seek purportedly life-saving treatments that were unsupported by scientific evidence, or even fraudulent. Desperate cancer patients have been targeted by sellers of “alternative” or quack drugs such as laetrile (Lerner, 1984), vitamin formulations, and many others. But many patients' access requests have involved medicines that were supported by some, albeit not conclusive, evidence, or medicines that were later approved for the condition. During the 1980s, AIDS patients mounted a highly publicized effort to gain early access to experimental antiretroviral drugs. In another well-publicized controversy in the 2000s, an advocacy group called the Abigail Alliance unsuccessfully sued the FDA for broader compassionate use after the founder's daughter was unable to enroll in clinical trials of two unapproved medicines for cancer, including a trial of a drug that was later approved for her particular diagnosis. More recently, compassionate use questions resurfaced in response to the Ebola epidemic, prompting an advisory panel to the World Health Organization to recommend that, in certain circumstances, it would be appropriate to treat patients with unapproved medicines (Hantel & Olopade, 2015).

The European Union (EU) and the USA, the world's largest pharmaceutical markets, have developed laws and policies that, in limited circumstances, permit access to experimental medicines and therapies before approval and outside of clinical trials. Their policies are based on similar principles and permit access only for terminally or seriously ill patients who do not have satisfactory therapeutic options among legally marketed treatments (European Medicines Agency, 2007; guideline on compassionate use of medicinal products, pursuant to Article 83 of Regulation (EC) No 726/2004) (U.S. Food and Drug Administration, 2014; Expanded Access to Investigational Drugs for Treatment Use, 74 Federal Register 40900–40945). Likewise, both the European Court of Human Rights and US courts have rejected terminally and seriously ill patients' claims that they possess an unfettered right to access unproven medicines (Hristozov and others v. Bulgaria, 2013, nos. 47039/11 and 358/12; http://hudoc.echr.coe.int/sites/eng/pages/search.aspx?i=001-114492) (Durisotto v. Italy, 2014, no. 62804/13; http://hudoc.echr.coe.int/sites/eng/pages/search.aspx?i=001-148030) (Shah & Zettler, 2010).

Although the general principles are similar, how these are implemented differs among EU member states, and between the EU and the USA (European Medicines Agency, 2007; guideline on compassionate use of medicinal products, pursuant to Article 83 of Regulation (EC) No 726/2004) (U.S. Food and Drug Administration, 2014; Expanded Access to Investigational Drugs for Treatment Use, 74 Federal Register 40900–40945) (Whitfield *et al*, 2010). Nonetheless, under many regulatory schemes—including Italy's—access to Stamina's stem cell treatments should not have been granted. Italy permits compassionate use of experimental cell therapies only when there are some published data justifying their use (Italian Ministry of Health, Decree December 5, 2006, published in the Official Journal March 9, 2007). In 2012, the Agenzia Italiana del Farmaco (AIFA, the Italian medicines agency) determined that this condition, among others, was not met and denied compassionate use. However, public pressure for access to the therapy was intense. Many Italian courts granted patients' access to it and Italian politicians provided funding for a clinical trial.

who makes decisions about patients' access requests can play an important role in the outcome of those requests.

The Stamina stem cell controversy is not an indictment of Italy's formal policy for compassionate use. After all, it was judges and politicians, not the AIFA, who decided to grant patients' access to the Stamina stem cell therapy despite the lack of evidence supporting its use. Instead, the controversy shows that *who* makes decisions about patients' access requests can play an important role in the outcome of those requests. When patients' requests are sympathetic, as they often are, and the public's hopes for an experimental medicine are high, it may be difficult for judges and politicians to deny requests even if they lack scientific merit. While expert regulatory agencies—such as the AIFA, the EMA, and the FDA—are not immune to such difficulties, they may be better situated than judges or politicians to determine whether the evidence suggests that a medicine, although unproven, is promising enough that granting access for particular patients is appropriate.

This, however, is not to say that formal expanded access policies are irrelevant or cannot be improved. In the 2000s, the FDA's expanded access rules were criticized for being vague and inconsistently applied. In response, the FDA revised its rules in 2009 to include specific criteria and requirements that must be met to authorize access, such as that the potential risks of the experimental medicine are reasonable in the context of the patient's disease (U.S. Food and Drug Administration, 2009; Expanded Access to Investigational Drugs for Treatment Use, 74 *Federal Register* 40900-40945). Likewise, it may be useful to clarify the standard for access to experimental medicines, including cell therapies, in EU member states by adding more specific criteria and requirements to help ensure that patients, companies that are asked to supply the medicines, and regulators share an understanding about which access requests are likely to be granted.

But even where regulatory agencies make decisions based on clear rules, those rules might not address all aspects of compassionate use. For example, the FDA authorizes the vast majority of the requests that it receives, but it cannot compel a company to provide a medicine; the decision about whether to provide a drug often ultimately falls to the company, which may have little guidance about how to prioritize access requests if, for example, the supply of the drug is limited. To address this dilemma, Johnson & Johnson recently created a panel of doctors, bioethicists, and patient representatives to advise it in its access decisions (Rockoff, 2015). If this proves useful, more companies may follow suit, or regulatory agencies may consider providing companies such guidance themselves.

Moreover, improving communication about experimental medicines may be of particular relevance for regenerative medicine because of the public's high hopes for the field (Kamenova & Caulfield, 2015). Indeed, a February 2015 report, issued in the wake of the Stamina controversy and spearheaded by Italian scientist and senator Elena Cattaneo, recommended that Italy provides its media with guidelines on communicating scientific information. But depending on the jurisdiction, there will be limits on how extensively the government can steer or influence media coverage about experimental medicines, because of constitutional protections for speech in democratic societies.

social media may play an increasingly important role in the debates.

The debate about expanded access/compassionate use is likely to continue for the foreseeable future, and new challenges will arise. More patients seem to be requesting expanded access—the FDA received almost double the number of access requests in 2014 as it did in 2013—and social media may play an increasingly important role in the debates as it gives patients and their advocates an effective tool to directly and publicly reach companies and regulators. Efforts to improve the compassionate use process outside of regulatory structures by, for example, improving communication about unproven medicines or establishing corporate advisory panels may prove useful in and of themselves, but perhaps more importantly, will help to identify new directions for refining laws and policies.

## Conflict of interest

The author served as an attorney in the U.S. Food and Drug Administration's Office of Chief Counsel from 2009 to 2013.
